# External beam radiotherapy for unresectable hepatocellular carcinoma, an international multicenter phase I trial, SAKK 77/07 and SASL 26

**DOI:** 10.1186/s13014-016-0745-0

**Published:** 2017-01-13

**Authors:** Evelyn Herrmann, Diana Naehrig, Manfred Sassowsky, Martin Bigler, Jeroen Buijsen, Ilja Ciernik, Daniel Zwahlen, Alessandra Franzetti Pellanda, Andreas Meister, Peter Brauchli, Simona Berardi, Erika Kuettel, Jean-François Dufour, Daniel M. Aebersold, for the Swiss Group for Clinical Cancer Research (SAKK)

**Affiliations:** 1 Department of Radiation Oncology, Inselspital, Bern University Hospital and University of Bern, Bern, Switzerland; 2 Division of Radiation Oncology, Basel University Hospital, Basel, Switzerland; 3 Department of Radiation Oncology, Lifehouse at RPA, Sydney, NSW Australia; 4 Department of Radiation Oncology and Division of Medical Radiation Physics, Bern University Hospital, Bern, Switzerland; 5 SAKK Coordinating Center, Bern, Switzerland; 6 Department of Radiation Oncology (MAASTRO Clinic), GROW – School for Oncology and Developmental Biolog, Maastricht, The Netherlands; 7 Department of Radiation Oncology, University of Zurich, Zurich, Switzerland; 8 Department of Radiotherapy and Radiation Oncology, Dessau City Hospital, Dessau, Germany; 9 Department of Radiation Oncology, Hospital Graubuenden, Chur, Switzerland; 10 Radiation Oncology Department, Oncology Institute of Southern Switzerland, Bellinzona, Switzerland; 11 Radiotherapy Service, Clinica Luganese SA, Lugano, Switzerland; 12 Centre for Radiation Oncology, KSA-KSB, Kantonsspital Aarau, Aarau, Switzerland; 13 Department of Hepatology, University Clinic of Visceral Surgery and Medicine, Inselspital, Bern University Hospital and University of Bern, Bern, Switzerland

**Keywords:** Hepatocellular carcinoma, Conformal radiotherapy, Liver, Radiation toxicity

## Abstract

**Purpose:**

To assess feasibility and safety of conventionally fractionated radiotherapy (cfRT) in patients with hepatocellular carcinoma (HCC).

**Methods:**

Patients with histologically confirmed stage cT1-4, cN0-1 HCC and Child-Pugh Score (CPS) A or B disease were included in a phase I multicenter trial. Metastatic HCC were allowed if ≥90% of total tumor volume was located within the liver. Patients were enrolled onto five dose-escalation levels (54–70Gy in 2Gy fractions) based on a modified 3 + 3 design, with cohorts of five patients instead of three patients in dose levels 4 and 5. Primary trial endpoint was dose-limiting toxicity (DLT), as specifically defined for 17 clinical and nine laboratory parameters as grade ≥3 or ≥4 toxicity (CTCAE vs. 3). The threshold to declare a dose level as maximum tolerated dose (MTD) was defined as a DLT rate of ≤16.7% in dose levels 1–3, and ≤10% in dose levels 4–5. Best objective response of target liver lesions and adverse events (AE’s) were assessed as secondary endpoints.

**Results:**

The trial was terminated early in DL 3 due to low accrual. Nineteen patients were recruited. Fifteen patients were evaluable for the primary and 18 for the secondary endpoints. Maximum tolerated dose was not reached. One patient in dose level 1, and one patient in dose level 2 experienced DLT (lipase > 5xULN, and neutrophils <500/μL respectively). However, dose level 3 (62Gy) was completed, with no DLTs in 3 patients.

Overall, 56% of patients had a partial response and 28% showed stable disease according to RECIST. No signs of radiation induced liver disease (RILD). Two patients in dose level 3 experienced lymphocytopenia grade 4, with no clinical impact.

**Conclusion:**

Conventionally fractionated radiotherapy of 58Gy to even large HCC was safe for patients with CPS A and B. 62Gy was delivered to three patients without any sign of clinically relevant increased toxicity. The maximum tolerated dose could not be determined.

**Trial registration:**

ClinicalTrials.gov identifier NCT00777894, registered October 21st, 2008.

## Background

Hepatocellular carcinoma is the most common primary liver tumor and the 2^nd^ leading cause of cancer related mortality worldwide. It represents 7% of all diagnosed cancers and its overall 5-year survival rate < 12%. In Europe, liver cirrhosis patients progress to HCC at a conversion rate of approximately 3% per year in Europe [1]. Prior to intensity-modulated radiotherapy (IMRT) or stereotactic body radiotherapy (SBRT), use of external beam radiotherapy (RT) has been quite limited in the treatment of HCC [2]. The whole liver has a low tolerance to radiation and patients are at risk for unacceptable liver toxicity [3]. Radiation injury to the liver after conventionally fractionated radiotherapy (cfRT), was first described by Ingold et al. several decades ago [4]. The clinical scenario of radiation induced liver disease RILD consists of anicteric hepatomegaly, ascites, and elevated alkaline phosphatase [5]. In the landmark report by Emami et al. [3], the whole-liver tolerance dose (TD) expected to yield a 5% risk of liver failure 5 years after treatment (TD 5/5) for whole-liver radiation was estimated to be 30Gy in 2Gy fractions. The Lyman normal tissue complication probability (NTCP) model and a local damage-organ injury NTCP model later have been used to describe the partial tolerance of the liver to RT [6]. Image-guided volumetric arc therapy (VMAT), IMRT and SBRT dose delivery, allow reducing the dose to non-tumor liver tissue, kidneys and the intestines. Thus, dose escalation to the diseased liver segments has become possible. This trial was conducted to obtain better understanding of the RT dose-response-relationship for tumor control as well as for normal tissue toxicities in this patient group. We report the results of the Swiss Group for Clinical Cancer Research (SAKK) 77/07 phase I trial assessing feasibility and safety of cfRT in patients with locally advanced non-resectable HCC.

## Patients and Methods

### Patients

Patients with unresectable, histologically or radiologically confirmed stage cT1–4, cN0–1 HCC, Barcelona clinic liver cancer (BCLC) stage B and C with no prior malignancy within 5 years were eligible for this phase I trial. Patients had to be older than 18 years, with a Child-Pugh A or B score, with a residual liver volume (= total liver volume – the gross tumor volume (GTV)) of >800 ml and ≥40% of uninvolved liver and WHO performance status 0–2. Metastatic HCC was allowed if ≥90% of total tumor volume was located within the liver. Exclusion criteria included, ALT and AST ≥5x upper limit of normal (ULN), AP ≥ 5 x ULN, bilirubin ≥3 x ULN, hemoglobin ≤ 100 g/L, neutrophils ≤1.2 x 10^9^/L, platelets ≤ 50 x 10^9^/L, international normalized ratio (INR) >2, creatinine clearance ≤ 50 mL/min, clinical ascites, encephalopathy, active hepatitis, gastric, duodenal, or variceal bleeding or weight loss ≥ 15% within three months of registration or esophageal varices ≥ grade 3 [7, 8]. Patients were also excluded, if they had prior RT to the abdomen or caudal chest below T5, prior transarterial chemoembolization (TACE) and radiofrequency ablation (RFA) within eight weeks. No chemotherapy was permitted within three weeks before registration. Portal vein thrombosis (PVTT) was not an exclusion criterion.

The study was planned and conducted in accordance with the principles of the Declaration of Helsinki. The protocol was approved by the ethics committee of each participating site (ClinicalTrials.gov identifier NCT00777894, registered October 21st, 2008). Written informed consent was obtained from all patients.

### Radiation therapy

Pretreatment triphasic diagnostic computed tomography (CT) was used to delineate the gross tumor volume (GTV) and enhanced area of vessel thrombosis. Patients were treated in supine position. Respiratory gating or breath holding techniques were used. Triphasic CT simulation with intravenous contrast was used with a 3- to 5-mm-slice thickness. The range of the simulation CT scan included the whole liver, lower part of the lungs, and both kidneys. The GTV was defined as the visible tumor on the arterial phase of simulation CT and/or fused diagnostic CT. For patients with coexisting PVTT, the area of PVTT was included as a part of the GTV. The clinical target volume (CTV) encompassed the area of the GTV with a 5–10 mm margin. The CTV was expanded by a 5- to 10-mm radial margin and a 6- to 15-mm cranio-caudal margin to create the planning target volume (PTV). Normal liver was defined as the whole liver volume minus the PTV. Patients were treated with either 3D-conformal RT (3D-CRT), IMRT or fractionated stereotactic RT techniques, using 6–18 MV photon energies. Due to compatibility between centers, dose prescription and normalization were fixed at isocenter and for conformal therapy, according to the international commission on radiation units and measurements (ICRU) reports 50 and 62. In case of IMRT, dose prescription and normalization could be defined as mean dose to PTV for optimization reasons, but for reporting it was rescaled to the isocenter. No normalization/prescription to isodose levels was allowed. For 3D-conformal planning: 2 to 5 portal beams with planar or non-coplanar arrangement were used. Larger number of fields was allowed to use to improve the quality of dose distribution [9]. For adequate target coverage, the PTV received 95–107% of the prescribed total dose. Alternatively, a minimum of 95% of the prescribed dose had to encompass more than 99% of the PTV. In addition, it was recommended that areas receiving more than 105% of the prescribed dose, are kept to <1% of the PTV. Our protocol suggested that participants have >800 mL and > 40% of non-tumor liver and that the mean dose has to be kept < 28Gy in patients with a non-cirrhotic liver and < 24Gy in patients with signs of cirrhotic liver. If the PTV encompassed >66% of the total liver volume, radiotherapy was not initiated. RT was delivered from Monday to Friday in five fractions per week, of 2Gy per fraction to a total dose of 10Gy per week. Normal tissue dose delivery guidelines for bowel, stomach, esophagus (each Dmean < 40Gy, V60Gy <3%), lung (V5Gy <85%, V20Gy <20% and Dmean < 12–15Gy), heart (Dmean < 40Gy), spine (D1% <48Gy) and kidneys (Dmean left kidney <12Gy and if V50% of right kidney irradiated >20Gy, Dmean left kidney <5Gy) were provided to facilitate planning [10, 11].

### Escalation strategy

Patients were to be enrolled onto five dose-escalation levels (54, 58, 62, 66 and 70Gy in 2Gy fractions, corresponding to a biological effective dose (BED) of 162, 174, 186, 198 and 210Gy with an alpha/beta ratio of 10, based on a modified 3 + 3 design. In dose levels 1 to 3, three patients, and in dose level 4 and 5, five patients had to be treated per cohort. Escalation to the next dose level was only permitted once no DLT was established within one month after the end of RT. If toxicity occurred in dose levels 1–3, a minimum of six patients and for dose level 4 and 5, a minimum of ten patients needed to be treated at that level, before escalating to the next level. While waiting until one month after RT, at which time the presence or absence of toxicity was determined, subsequent patients could be treated at the pre-defined dose level, up to a dose of 44Gy. DLT was specifically defined for 17 clinical and nine laboratory parameters as grade ≥3 or ≥4 toxicity according to CTCAE vs. 3. The threshold to declare a dose level as MTD, was defined, as a DLT rate of ≤16.7% in dose levels 1–3, and ≤10% in dose levels 4–5. A dose of 54Gy was chosen as the lowest dose level, because doses up to 54Gy showed response rates at around 50% [12].

### Quality assurance (QA)

Before starting patient accrual, each participating center was required to successfully participate in a radiotherapy specific quality assurance (RT-QA) program corresponding to EORTC QART [13] levels 1 and 2: 1.) Facility questionnaire External dosimetry audit (EDA), 2.) dummy run (DR). For the DR an anonymized case including CT data set was made available. Target volumes (GTV, CTV, PTV) and all organs at risk (OAR) were delineated by the center’s investigator and reviewed by the coordinating investigator. Deviations from the protocol were communicated to the participating center, and a revised version of the delineated structures was requested and reviewed. The approved structures were then used to elaborate a treatment plan using the RT technique chosen by the center. The plans were reviewed by the trial chair and trial medical physicist, which requested a revised version, in case of deviations from the protocol.

An internal report comparing the anonymized DR results of the first five participating centers was made available to those centers. Coincidence histograms [14] were used for a quantitative topological comparison of the delineated structures.

### Evaluation

Patients were assessed weekly during RT, and after completion of treatment, followed up for one year at 1, 2, 3, 5, 8 and 11 months. At the first month follow-up physical examination, hemoglobin, neutrophils, platelets, hepatic function, renal function, pancreatic lipase, INR and CPS were reviewed. The reason for including pancreatic lipase in the follow up examination was to discover pancreatitis. Liver triphasic CT was performed at 2 and 5 months after RT, thereafter every 3 months until progression. At each follow-up within the first three months after RT, toxicity was graded using the CTCAE vs. 3. RILD was defined as the development of nonmalignant ascites without disease progression and an anicteric elevation of alkaline phosphatase level by at least twofold. Non – classic RILD was defined as the development of jaundice and/or elevated serum transaminases (>5 x UL) within 3 months of completion of RT in patients with underlying chronic hepatic disease (cirrhosis or viral hepatitis). Best objective response of target liver lesions was assessed using RECIST.

### Statistics

Using a modified 3 + 3 design, the planned sample size laid between two and 38 evaluable patients in case of two DLTs in the first two patients and two full cohorts at each dose level, respectively. For the primary endpoint, the following patients were considered evaluable: either patients who experienced a DLT and received a dose of at least 12Gy or patients who completed the treatment according to their dose level. Non-evaluable patients were to be replaced. Primary endpoint was DLT. Best objective response of target liver lesions and adverse events were assessed as secondary endpoints. Local control was defined as time from registration to progression of target lesions or death due to progression, whichever occurred first. Overall survival was the time from registration to death from any cause. The rates at one year of these survival times were estimated using the Kaplan-Meier estimator. Point estimates and, if applicable, the corresponding exact 95% confidence interval were calculated for proportions. Median follow-up time was calculated using the reverse Kaplan-Meier method. All data were collected and analyzed at the SAKK coordinating center in Bern, Switzerland. Analyses were performed using SAS 9.2.

## Results

The trial was terminated early due to low accrual. Therefore only dose levels 1–3 (54, 58 and 62Gy) were examined.

### Patients

From November 2008 to January 2014, 19 patients from five centers within Switzerland and the Netherlands were recruited; six patients at dose level 1, seven patients at dose level 2 and six patients at dose level 3. One patient was not treated at all with RT because of inadequate normal liver volume < 800 ml and therefor not evaluable for both endpoints.

In DL 3, three additional patients were not evaluable for the primary endpoint: two died during treatment one due to cardiac failure and one due to pneumonia (at 26 and 30Gy in 2Gy per fraction, respectively), and one because laboratory values were repeatedly not measured, making it impossible to assess DLT. The remaining 15 patients completed RT as planned, and were evaluable for the primary endpoint. For the secondary endpoints, 18 patients (95%) were evaluable. Median follow up time was 11.8 months. The median age was 68 years (range 45–82 years, inter-quartile range 62–77). The majority of HCC patients had stage cT3 (47%), cN0 (84%) disease, CP-score A5 (58%) and WHO performance status 0 (53%). Alcohol was the most common etiology of the underlying liver cirrhosis (74%). Two patients (11%) had M1 disease. Median longest diameter of largest lesion was 68 mm (range 18–230 mm). Median administered RT dose was 57Gy (range 26–62Gy in 2Gy per fraction). Other baseline patient characteristics can be found in Table 1. Median longest diameter of largest lesion was 70 mm (range 18–185 mm). Median administered RT dose was 57Gy (range 26–62Gy in 2Gy per fraction).

**Table 1:** Patient characteristics of all registered patients (*n* = 19)

Variable	Value (*N* = 19)
Age [years]	
Median (Min–Max), *N* = 19	68 (45–82)
Gender	
F	3 (16%)
M	16 (84%)
T stage	
1	2 (11%)
2	4 (21%)
3	9 (47%)
4	4 (21%)
N stage	
0	16 (84%)
1	3 (16%)
M stage	
0	17 (89%)
1	2 (11%)
Total Child-Pugh score	
5	11 (58%)
6	4 (21%)
7	3 (16%)
8	1 (5%)
WHO performance status	
0	10 (53%)
1	7 (37%)
2	2 (11%)
BCLC classification	
0	1 (5%)
A	1 (5%)
B	10 (53%)
C	7 (37%)
Vascular invasion	
Yes	8 (44%)
No	9 (50%)
Unknown	1 (6%)
Missing	1
Previous therapies (more than 1 possible)	
TACE	2 (11%)
TAE	1 (5%)
PEI	0
Local thermal ablation	1 (5%)
Liver resection	2 (11%)
Systemic treatment	3 (16%)
Other	0
Longest diameter of largest lesion [mm]	
Median (Min–Max), *N* = 19	86 (18–230)
Gross tumor volume [ml]	
Median (Min–Max), *N* = 19	340 (10–3582)
Total liver volume [ml]	
Median (Min–Max), *N* = 19	2136 (948–3400)
Residual liver volume [ml]	
Median (Min–Max), *N* = 19	1630 (906–2283)
Etiology of underlying liver disease (more than 1 possible)	
Hepatitis B	1 (5%)
Hepatitis C	2 (11%)
Alcohol	14 (74%)
Unknown	3 (16%)

### Toxicity

At dose levels 1 and 2, six patients each and at dose levels 3, three patients were evaluable. One (17%) of 6 patients in dose level 1 and one (17%) of six patients in dose level 2 experienced DLTs (lipase > 5xULN and neutrophils <500/μL). Both, dose levels 1 and 2 had a DLT each among the first three patients, requiring an additional three patients at the same dose level as per protocol. However, dose level 3 (62Gy) was completed, with no DLTs in three patients. Two patients died during RT treatment, one patient in dose level 2 due to cardiac failure, and one in dose level 3 due to pneumonia. One patient in dose level 3 died during follow-up, due to cardiac failure. None of the deaths were related to RT treatment. None of the in total evaluated patients showed any signs of RILD. At dose level 1, one patient developed an elevated lipase value grade 4 (Lipase > 5 x ULN) during the first week of RT, which subsequently normalized on laboratory follow-up. A second patient showed an elevated grade 4 bilirubin value (Total bilirubin > 10 x ULN) one month after RT, which normalized, and a third patient showed an isolated elevated AST grade 4 (AST > 20 x ULN) three months after RT, which also normalized during the subsequent analyses. At dose level 2, one patient experienced neutropenia grade 4 (neutrophils <500/μL) in week 6 of RT and at dose level 3, two patients each experienced lymphocytopenia grade 4 (lymphocytes < 200/mm^3^) during week 5 and 6 as well as during week 6 and at the end of RT, with no clinical impact.

### Response rate

**Fig. 1:**
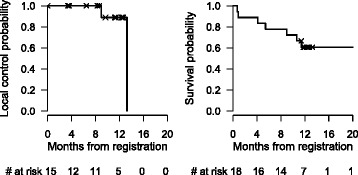
Kaplan-Meier curves for local control and overall survival

The overall RECIST response rate was 56% (95% CI 31–78%), with 0% complete response (CR), 56% partial response (PR) and 28% stable disease (SD). In three patients (16%), treated at dose level 3, no response (NR) could be determined. Two patients died and one had symptomatic deterioration before their first CT scan after baseline. An overview of response rate according to DL and CP-Score are shown in Tables 2 and 3. The most frequent site of first progression was outside the treated volume. One-year survival and 1-year local control in this small cohort is 61% (95% CI: 35–79%) and 89% (95% CI 43–98%), respectively, which were not endpoints within the trial (Fig. 1).

**Table 2:** Overall response of target lesions according to RECIST v1.0 by DL

	Overall	DL 1 (54Gy)	DL 2 (58Gy)	DL 3 (62Gy)
Best response (N)	18	6	6	6
PR	10 (56%)	4 (67%)	4 (67%)	2 (33%)
SD	5 (28%)	2 (33%)	2 (33%)	1 (17%)
NA	3 (17%)			3 (50%)

**Table 3:** Overall response of target lesions according to RECIST v1.0 by CPS

	CPS A	CPS B
Best response (N)	14	4
PR	8 (57%)	2 (50%)
SD	4 (29%)	1 (25%)
NA	2 (14%)	1 (25%)

## Discussion

We present the data of an international multicenter phase I study of 18 patients treated with cfRT for locally advanced non-resectable HCC. In contrast to previously published prospective studies [10, 15–18], DLT in the present trial was specifically defined for 17 clinical and nine laboratory parameters as grade ≥3 or ≥4 toxicity (CTCAE vs. 3), to address the safety aspect from a biochemical point of view. Additionally, our study-population had large tumors (median 86 mm, range 18–230 mm) and 20% of patients had a CP B score. Even in such a vulnerable patient collective, the present trial showed that cfRT of 58Gy to HCC is safe in an international multicenter setting. A total dose of 62Gy was delivered to three patients without any sign of clinical relevant increased toxicity. However, the maximum tolerated dose could not be determined due to the early termination of the trial because of patient accrual. The reasons for very slow patient accrual included several competing focal treatment options (e.g. RFA, TACE) in liver tumors and the rapid evolvement of SBRT. RT was well tolerated in this study, and no signs of RILD were observed. The DLTs at dose level 1 (54Gy) and 2 (58Gy) (lipase > 5xULN and neutrophils <500/μL) were not clinically relevant. The increased lipase value occurred in the first week of RT at DL 1 and was most likely not related to RT, since it normalized in the subsequent weeks of treatments, when higher cumulative doses were applied. The decreased granulocyte value may have corresponded to an increased granulocyte consumption, which may be interpreted as an expression of intermittent hepatocyte injury [19]. During follow-up, there was a spontaneous remission of the neutrophils in the physiological range. Therefore it is questionable to use the lipase and neutrophils as a dose-limiting factor for RT induced liver injury. The isolated bilirubin elevation one month after RT and isolated AST elevation at 3 months after RT were only present at dose level 1. No patient in dose level 2 and 3 had similar laboratory changes, even though higher doses were applied. The grade 4 adverse events were transient, and all patients recovered spontaneously within a few months. None of the two patient’s deaths in this trial were related to RT treatment.

Several studies showed that it is safe to treat HCC patients with cfRT in HCC. In a series of prospective trials [10, 15–18], the University of Michigan group first established the safety of an individualized dose allocation approach for liver cancer. They developed a NTCP model that quantitatively described the relationship between dose and volumes irradiated and the probability of developing classic RILD using conformal RT techniques. Radiation dose was individualized based on the volume of normal liver that could be spared without exceeding a 5–20% risk of RILD. Objectively measurable disease was not an entry criterion, although it was followed when available. The prescribed doses ranged from 40–90Gy (median, 60.75Gy) in 1.5Gy twice-daily fractions delivered with concurrent hepatic arterial fluorodeoxyuridine [2]. In a phase II trial, Ben-Josef et al. [10] reported median survival of 15.8 months with a trend to improved survival (23.9 vs. 14.9 months) in patients treated with doses of ≥75Gy. Doses below 60Gy had little effect on survival and then a steady increase in survival was observed as RT dose increased to 90Gy. Of the 128 patients, 30% patient developed mostly biochemical grade 3 to 4 toxicity, five patients (4%) developed RILD. In a large retrospective series from Korea [20] including 158 HCC patients with CPS A or B were treated with 25–60Gy in 1.8Gy daily fractions. The patient selection was similar to the one of the present trial. Median overall survival time was 10 months, with no grade 4 or 5 toxicity reported. They demonstrated that the CP score was a significant factor in the development of RILD and the total radiation dose was the only significant factor determining the tumor response. The same group [21] reported in a retrospective patterns of care study of 398 patients, with HCC treated at 10 institutions in Korea, that CPS A, tumor size <5 cm, negative lymph nodes and BED > 53.1Gy _(_alpha/beta of 10) were significant factors for a better prognosis. In their collective BEDs between 4.2–124.3Gy were delivered, the median survival time was 12 months, and the 2-year overall survival rate was 27.9%.

For that reason we suggest, that dose escalation, exceeding 62Gy, should be chosen based on the NTCP of the surrounding liver tissue. One-year survival and 1-year local control in our trial with this vulnerable cohort were 61% and 89%, respectively, although they were not endpoints in the present study.

The overall RECIST response rate in our trial was 56%, with no patient showing a CR. Mornex et al. [22] showed in their phase II trial, including 27 patients, a 92% response rate using the WHO and RECIST 1.0 criteria. Ninety-six percent of patients received an RT dose of 66Gy. However, they only included patients with small-size HCC between ≥30 and ≤50 mm, whereas in our trial, tumor size ranged from 18–230 mm. Of all patients, 41% developed grade ≥3 toxicity: 19% asymptomatic grade 3 laboratory parameters toxicities in CPS A patients and 27% grade 4 laboratory parameters toxicities, 15% late grade 3 toxicity consisting of gastric bleeding requiring transfusion, and edematous-ascitic hepatic decompensation requiring paracentesis and diuretics in CPS B patients.

Liu et al. [23] treated 44 patients with large HCC (60–250 mm) with 40–60Gy in standard fractionation. Tumor response was based on serial CT scans, with an overall response rate of 61% using the WHO response rating criteria. Radiation-induced toxicities remained mild and reversible. Their results are comparable with the results of our study. Similarly, a retrospective study of Toya et al. [24] treated 38 HCC patients with PVTT and tumor sizes ranging from 9 to 93 mm. A total dose of 17.5–50.4Gy (median 40Gy), in 1.8–4Gy per fraction was delivered, which translated to a BED of 23.4–59.5Gy (median 50.7Gy) with an alpha/beta of 10. Response rate was 44.7%. In 13 patients treated with 45Gy in 3Gy per fraction, the response rate was 76.9%. The PVTT size (≤30 mm vs. ≥ 30 mm) and BED ≥58Gy _(_alpha/beta of 10) were factors, which significantly were influencing response rate and survival. The median- and one-year survival was 9.6 months and 39.4%, respectively.

However, using the RECIST criteria to evaluate RT response rate is outdated. It has been shown that extensive tumor necrosis after loco-regional ablative treatment or systemic chemotherapy may not always be followed by an overall reduction in tumor diameter. In some instances the lesion size may even increase due to necrosis [25]. Several recent studies [26–30] have demonstrated that quantification of residual viable tumor by the European Association for Study of the Liver (EASL) and modified RECIST (mRECIST) guidelines better predict treatment response compared with WHO and RECIST guidelines. Volumetric response assessment is likely to become the gold standard for defining treatment response [31, 32]. Our protocol was written during a period of time, when the mRECIST guidelines were not standard yet. The available literature on response rates after cfRT reports in WHO or RECIST criteria.

The presented data is well comparable with the existing data within the literature. However, it has its limitations. It is a small number of patients and the maximum tolerated dose could not be determined due to the early termination of the trial. Also the trial duration was long. During this time period other effective treatment techniques such as SBRT or proton beam therapy have evolved. SBRT refers to the use of stereotactic non-coplanar conformal radiation therapy to precisely deliver a large ablative radiation dose in a small number of fractions, while limiting the dose to adjacent normal tissues. The steep dose gradient within the target volume leads to tight conformity with steep and isotropic dose fall-off and high dose delivery to the target volume [33]. There is a growing SBRT experience, mostly in patients with small (<6 cm) HCC [34–38] with a high local control ranging from 70–90% at one and two years. In a large Canadian phase I/II study by Bujold et al. [39], 102 patients with locally advanced HCC (median size, 10 cm) were treated with six fractions of SBRT, with a 1-year local control rate of 87% and median OS of 17 months. Despite limiting their study to a CP A score population, CP class deterioration occurred in 29% at 3 months. Proton radiotherapy has also emerged as a treatment option for patients with localized HCC. It enables further dose escalation and precise dose delivery while maintaining a favorable toxicity profile. Various phase II trials have demonstrated the effectiveness and toxicity profile of this therapy [40–42].

Recent studies have compared SBRT and proton beam therapy to other focal treatment options such as RF or TACE [43–45] in early stage HCC patients. Wahl et al. [43] have published a retrospective study comparing SBRT to RF in inoperable patients with small HCC. For tumors treated with RFA, freedom from local progression (FFLP) at two years was 80.2% vs. 83.8% for SBRT. Increasing tumor size was predictive for FFLP in patients treated with RFA (hazard ratio [HR], 1.54 per cm; *p* = 0.006), but not for those treated with SBRT (HR, 1.21 per cm; *p* = 0.617). For tumors ≥2 cm, there was decreased FFLP for RFA compared with SBRT (HR, 3.35; *p* = 0.025). Takeda et al. [44] conducted a phase II study, treating 90 CP A and B score patients with a solitary HCC lesion up to a diameter of 4 cm, unsuitable for resection and RF with SBRT and optional TACE. Three-year LC rate and OS was 96.3% and 66.7% (95% CI, 56.3–75.6%) respectively. In an other phase II study Bush et al. [45] compared proton beam therapy to TACE as a bridge for transplantation. In an interim analysis of 69 subjects, ten TACE and 12 proton patients underwent liver transplantation after treatment. Viable tumor identified in the explanted livers after TACE/proton averaged 2.4 and 0.9 cm, respectively. Pathologic complete response after TACE/proton was 10%/25% (*p* = 0.38). The two-year OS for all patients was 59%, with no difference between treatment groups. Median survival time was 30 months (95% CI 20.7–39.3 months). There was a trend toward improved two-year LC (88% vs. 45%, *p* = 0.06) and progression-free survival (48% vs. 31%, *p* = 0.06) favoring the proton beam treatment group.

However, large, inoperable HCC > 10 cm, remain challenging for treatment, because of close proximity to critical organ, limited liver volume available and a relatively poor liver functional status. In this small niche of treatment indications, when locally ablative treatments like RFA, TACE or SBRT are not possible, cfRT remains a valid treatment approach for liver cancer [46]. Conventional fractionation schedules may be more robust for certain patients with large tumors or at risk for fibrosis of the biliary ducts. The relatively high alpha/beta ration of 8 [47] of liver tissue implies highly conformal therapy, if treatment is completed within a few sessions. Nevertheless, the use of SBRT is being preferred whenever feasible. The steep dose gradient within the target volume leads to tight conformity with steep and isotropic dose fall-off and high dose delivery to the target volume and requires, due to the complementary information, when ever available the addition of MRI imaging to GTV delineation as well as appropriate motion control [48].

Despite increasing utilization, and prospective phase II studies [39] describing favorable outcomes, SBRT for liver cancer is still not included in practice guidelines [49–51]. There is currently one randomized phase III trial by the RTOG (RTOG 1112, (ClinicalTrials.gov ID: NCT01730937) open for accrual, comparing Sorafenib versus SBRT followed by Sorafenib in locally advanced HCC. This trial hopefully will help to better clarify the role of RT in HCC.

## Conclusion

This multicenter trial showed that conventionally fractionated radiotherapy delivering 58Gy to large primary tumors of the liver was safe for patients with CPS A and B. The dose of 62Gy was delivered to three patients without any sign of increased toxicity. However, the maximum tolerated dose could not be determined due to the early termination of the trial. Randomized trials are warranted to further define the role of cfRT and SBRT within multimodal treatment concepts for unresectable HCC.

## Abbreviations

3D-CRT: 3D-conformal RT
BED: Biologically effective dose
cfRT: Conventionally fractionated radiotherapy
CPS: Child-Pugh score
CR: Complete response
CT: Computed tomography
CTCAE vs. 3: Common toxicity criteria for adverse events version 3.0
CTV: Clinical target volume
DLT: Dose-limiting toxicity
DR: Dummy run
EASL: European Association for Study of the Liver
EDA: External dosimetry audit
GTV: Gross tumor volume
HCC: Hepatocellular carcinoma
IMRT: Intensity-modulated radiotherapie
INR: International normalized ratio
mRECIST: Modified RECIST
MTD: Maximum tolerated dose
NR: No response
NTCP: Normal tissue complication probability
OAR: Organs at risk
PR: Partial response
PTV: Planning target volume
PVTT: Portal vein thrombosis
RECIST: Response evaluation criteria in solid tumors
RFA: Radiofrequency ablation
RILD: Radiation induced liver disease
RT: Radiotherapy
RT-QA: Radiotherapy specific quality assurance
SAKK: Swiss Group for Clinical Cancer Research
SBRT: Stereotactitc body radiotherapy
SD: Stable disease
TACE: Transarterial chemoembolization
TD: Tolerance dose
ULN: Upper limit of normal
VMAT: Volumetric arc therapy
